# Biocontrol Potential of Antagonistic Yeasts on In Vitro and In Vivo *Aspergillus* Growth and Its AFB_1_ Production

**DOI:** 10.3390/toxins15060402

**Published:** 2023-06-19

**Authors:** Dilara Nur Dikmetas, Hayrettin Özer, Funda Karbancıoglu-Guler

**Affiliations:** 1 Department of Food Engineering, Faculty of Chemical and Metallurgical Engineering, Istanbul Technical University, 34469 Istanbul, Türkiye; dikmetas@itu.edu.tr; 2 The Scientific and Technological Research Council of Türkiye (TÜBİTAK), Marmara Research Center (MRC), 41470 Gebze, Türkiye

**Keywords:** *Aspergillus* spp., aflatoxin, mycotoxin, biocontrol, antagonistic yeast, volatile organic compounds, hazelnut

## Abstract

*Aspergillus flavus* is a major aflatoxin B_1_, posing significant health concerns to humans, crops, and producer fungi. Due to the undesirable consequences of the usage of synthetic fungicides, biological control using yeasts has gained more attention. In this study, eight isolates of epiphytic yeasts belonging to *Moesziomyces* sp., *Meyerozyma* sp. and *Metschnikowia* sp., which have been identified as antagonists, were isolated from different plants, including grapes, blueberries, hawthorns, hoşkıran, beans and grape leaf. While volatile organic compounds (VOCs) produced by *Moesziomyces bullatus* DN-FY, *Metschnikowia* aff. *pulcherrima* DN-MP and *Metschnikowia* aff. *pulcherrima* 32-AMM reduced in vitro *A. flavus* mycelial growth and sporulation, only VOCs produced by *Metschnikowia* aff. *fructicola* 1-UDM were found to be effective at reducing in vitro AFB_1_ production. All yeasts reduced the mycelial growth of *A. flavus* by 76–91%, while AFB_1_ production reduced to 1.26–10.15 ng/g and the control plates’ growth was 1773 ng/g. The most effective yeast, *Metschnikowia* aff. *Pulcherrima* DN-HS, reduced *Aspergillus flavus* growth and aflatoxin B_1_ production on hazelnuts. The AFB_1_ content on hazelnuts reduced to 333.01 ng/g from 536.74 ng/g. To our knowledge, this is the first report of yeasts isolated from plants being tested as potential biological control agents to reduce AFB_1_ production on hazelnuts.

## 1. Introduction

Mycotoxins are toxic metabolites produced by fungi, particularly saprophytic molds that grow on agricultural products. They are harmful to human and animal health and cause economic losses and reduce crop quality [1]. Aflatoxin, a significant mycotoxin of concern in agriculture, is mainly produced by *Aspergillus flavus* and *Aspergillus parasiticus* [2]. Various crops, such as maize (*Zea mays* L.), groundnut (*Arachis hypogaea* L.), cottonseed (*Gossypium* spp.), pistachio (*Pistacia vera* L.) and almond (*Prunus dulcis* Mill.) are prone to contamination by aflatoxin-producing fungi under both pre and post-harvest conditions [3]. Aflatoxins can pose significant health concerns to humans and animals and have been identified as a serious food safety concern [4], as well as being classed as group 1 carcinogens by the International Agency for Research on Cancer [5]. Aflatoxins, which are grouped into 20 chemically related metabolites, are of four major types: aflatoxins B_1_, B_2_, G_1_ and G_2_ (AFB_1_, AFB_2_, AFG_1_ and AFG_2_) [6]. In Turkey, aflatoxin occurs most commonly on dried figs, pistachios, hazelnuts, and groundnuts [7]. Turkey’s national legislation on mycotoxins is in line with EU standards. The maximum levels in various nuts, grains and different fruits and spices are in the range of 2 μg/kg–12 μg/kg for AFB1 and 4 μg/kg–15 μg/kg for the sum of aflatoxins [8]. Crop contamination with aflatoxin has significant indirect and direct economic effects. The loss of produce or market value, medical expenses, and related costs are examples of the direct economic impact. Animal losses and the cost of preventing and monitoring food-borne illnesses are just two examples of indirect economic effects [1]. Due to crop losses brought on by mycotoxigenic fungi like *A. flavus*, these regulatory guidelines (applied both domestically and internationally) have placed a significant financial burden of over US$932 million on agriculture worldwide. Depending on the market, economic losses could reach 100% due to the product being completely rejected when aflatoxin levels exceed acceptable limits. Due to requirements to comply with EU standards for all food exports, Africa alone loses more than US$670 million annually [1].

Hazelnuts are a significant export for Turkey. According to Alasalvar, Amaral, and Shahidi [9], hazelnut kernels are very nutrient-dense due to their high concentrations of fats (particularly oleic acid), proteins, carbohydrates, dietary fiber, vitamins (especially vitamin E), minerals, tocopherols (α-tocopherol), phytosterols (β-sitosterol), polyphenols, and squalane. Hazelnut is one of the most widely grown nut crops worldwide, with Turkey and Italy leading the world in production (665,000 tons and 140,560 tons, respectively), with a market share that is constantly expanding [10]. The existence and expansion of aflatoxigenic fungi, which can result in the production of aflatoxins and the contamination of hazelnuts, is a significant obstacle to the hazelnut industry. Aflatoxigenic fungi can infect hazelnuts in the orchard before harvest, during harvest, and especially during storage after the shell has been cracked [11]. 

AFB_1_ is known as the most carcinogenic, mutagenic, and teratogenic substance that naturally presents in foods and feeds [12]. As a result, detecting and preventing contamination by *Aspergillus* species, as well as reducing the level of aflatoxins in grains used in many agricultural products, is critical [2]. In addition to political pressure to remove hazardous pesticides from the market, the use of chemical pesticides has been restricted [13]. Therefore, a better alternative is needed to manage and detect aflatoxin producers in their early stages of growth. To reduce synthetic fungicide application, biological control with microbial antagonists has emerged as a promising alternative due to being environmentally safe and sustainable [14]. Among bacteria, members of the genera *Bacillus* sp. can control *Aspergillus* sp. growth [15,16]. Applying competitive and non-aflatoxigenic strains of *A. flavus* and *A. parasiticus* to soil has been shown to be an effective technique for preventing pre-harvest aflatoxin contamination of crops among aflatoxin contamination management approaches by several studies [6,17,18]. 

Yeast has become more interesting due to some characteristics that could be used as biocontrol agents, including growing faster than fungal pathogens, simple nutritional requirements and the ability to colonize dry surfaces of several niches to compete for nutrients and space [19]. In addition, yeasts are recognized as harmless to humans in the absence of allergenic spore production [20]. Besides important characteristics, including their efficacy against phytopathogenic fungi, yeasts may become a promising alternative to synthetic fungicides among various microorganisms that have already been reported by several studies [14,21,22,23]. The management of fungal contamination by antagonistic yeasts has been described by several mechanisms such as competition for space and nutrients, biofilm formation, parasitism, production of diffusible antimicrobial compounds, antibiosis, lytic enzyme production, and production of antimicrobial volatile organic compounds (VOCs). The antifungal activity of the VOCs produced by yeast isolates was documented against *Botyrtis cinerea* [24,25]; *Aspergillus carbonarius* [23]; *Penicillium expansum* and *Penicillium digitatum* [26]. Recent studies have investigated the ability of antagonistic yeasts to inhibit or reduce Ochratoxin A (OTA) [27,28]. However, studies investigating yeasts’ and yeast VOCs’ abilities to control *A. flavus* growth and aflatoxin B_1_ production are limited. 

The antagonistic impact of *Metschnikowia* spp. on various molds has been extensively documented, including *Penicillium* spp. [20], *Alternaria* spp., *Aspergillus* spp., *Fusarium* spp. [29] and *Botrytis cinerea* [30]. Additionally, it is well documented that the antagonistic effect of *Meyerozyma guilermondii* (previously known as *Pichia guilermondii*) on different post-harvest pathogens significantly affects fruit and vegetables [31,32,33]. However, biocontrol ability is not a general characteristic of *Moesziomyces* (previously known as *Pseudozyma*), which is an environmental yeast commonly isolated from plant leaves, flowers and soil [20]. Recently, *M. pullcherima* was found to effectively control green mold disease on mandarin [34] and *Botrytis cinerea* infection in apples [35]. *M. guilermondii* was effective at controlling gray mold on kiwi fruit [36]. Parafati et al. [37] documented that *M. pulcherrima* could be considered a biocontrol agent for controlling *A. flavus* contamination on pistachio nuts. Few studies have focused on the decontamination of *Aspergillus* spp. or aflatoxins on hazelnuts using low-pressure (LP) [38], atmospheric-pressure (AP) plasmas [38,39], cold plasma [40] and diffuse barrier discharge [41]. However, based on our literature review, there is no information on the control of the growth of *A. flavus* or other molds and on AFB_1_ production on hazelnuts using a biocontrol agent. 

This study aimed to isolate and identify the yeasts, determine their biocontrol capabilities against the growth of *A. flavus* and its AFB_1_ production and evaluate the most effective antagonistic yeast for controlling *A. flavus* growth and AFB_1_ production on hazelnuts.

## 2. Results

### 2.1. Isolation and Molecular Identification of Yeast Isolates

Eight yeasts were isolated from hawthorn, bean leaves, corn tassel, and hoşkıran; four of them were selected and identified by PCR amplification and sequencing the 16s rDNA gene. In this study, four *Metschnikowia* aff. *pulcherrima*, one *Metschnikowia* aff. *fructicola*, one *Metschnikowia pulcherrima*, one *Meyerozyma guilliermond*ii and one *Moesziomyces bullatus* were used (Table 1).

**Figure 1:**
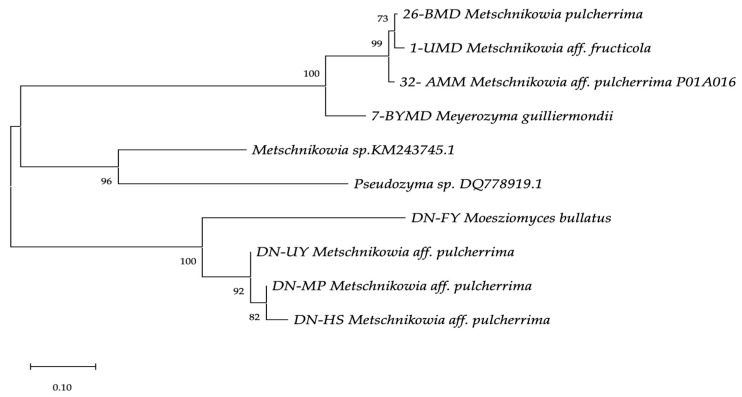
Phylogenetic tree of the yeast isolates generated using the neighbour-joining method. The percentage of replicate trees in which the associated taxa clustered together in the 1000 bootstrap replicates are shown next to the branches. The evolutionary distances were computed using the Maximum Composite Likelihood method. Branch lengths are proportional to nucleotide differences as indicated on the bar. Reference sequences were retrieved from GenBank under the accession numbers indicated. Evolutionary analyses were conducted in Mega X.

A phylogenetic tree was constructed with reference strains of *Metschnikowia* sp., *Meyerozyma guilliermondii* and *Pseudozyma* species using the neighbour joining method [43]. Phylogenetic distances were computed using the maximum composite likelihood method [44] implemented in MegaX [45]. The percentage of replicate trees in which the associated taxa clustered together in the 1000 bootstrap replicates are shown next to the branches. The evolutionary distances were computed using the Maximum Composite Likelihood method. Branch lengths are proportional to nucleotide differences, as indicated on the bar. Reference sequences were retrieved from GenBank under the accession numbers indicated. Evolutionary analyses were conducted in Mega X (Figure 1).

### 2.2. Antagonistic Activity on Agar Plates

**Figure 2:**
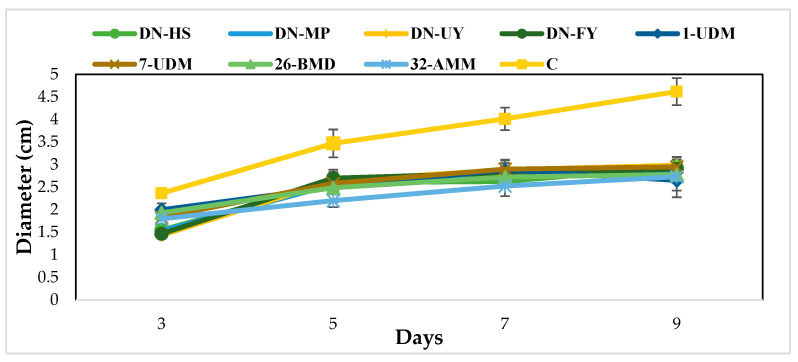
Biocontrol activity of eight yeast cultures (DN-HS, DN-FY, DN-MP, DN-UY, 1-UD7, UD26-BMD, 32-AMM) against *A. flavus*. Results are expressed as colony diameter (cm) over 9 days of incubation at 25 °C. Vertical bars indicate standard error (*n* = 6).

The dual culture assay was used for preliminary screening of all yeast isolates’ antagonistic activities against the aflatoxin B_1_ producer *A. flavus*. The results showed that all isolates inhibited the mycelial growth of *A. flavus.* Limited fungal mycelium growth and spore production inhibition were clearly visible in the zone surrounding the *Metschnikowia* aff. *pulcherrima* DN- HS colony, which indicated inhibition (Figure S1). Mycelial growth did not exceed or reach any yeast isolates. Compared to untreated control, the colony diameter was reduced by 36–40% after 9 days of incubation (Figure 2).

The colony diameters of cultures that interacted with *Moesziomyces bullatus* DN-FY, *Metschnikowia* aff. *pulcherrima* DN-MP, *Metschnikowia aff. fructicola* 1-UDM and *Metschnikowia pulcherrima* 26-BMD were statistically different on day 3 of incubation compared with other incubation periods (*p* < 0.05). However, control plate colony diameters were statistically different over the incubation period. Additionally, after 9 days of incubation, fungal colony diameters of all yeast isolates were statistically different from the control plates. Results are expressed as colony diameters (mm) over 9 days of the incubation period (Table 2).

### 2.3. Effects of Volatile Organic Compounds (VOCs)

According to studies, the production of antifungal VOCs by *M.* aff. *pulcherrima* DN-HS, *M. guilliermondii* 7-BYMD and *M. pulcherrima* 26-BMD had no significant impact on mycelium growth inhibition compared with control plates after 7 days of incubation. There were significant differences between *M. bullatus* DN-FY, *M.* aff. *pulcherrima* DN-MP and *M.* aff. *pulcherrima* 32-AMM on the 7th day compared with other yeast cultures (Table 3). *M. bullatus* DN-FY, *M.* aff. *pulcherrima* DN-MP and *M.* aff. *pulcherrima* 32-AMM isolates showed the highest antagonistic activity, with mycelial growth reduction of 12.49%, 11.8% and 11.07%, respectively. Additionally, higher inhibition rates were observed for *M. bullatus* DN-FY isolates during all incubation periods. However, mycelial growth inhibition rates were highest on day 3 of incubation for all of the yeast isolates. Volatile organic compounds’ effects decreased after the long incubation period.

**Figure 3:**
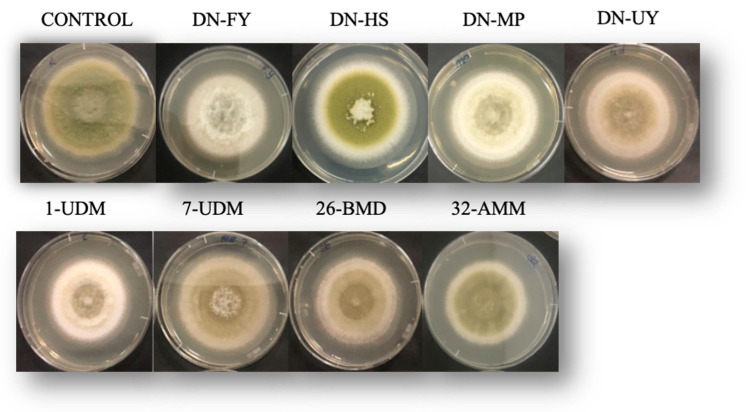
*A. flavus* cultured plates covered with VOCs produced by yeast cultures after 7 days of incubation.

Morphological changes were observed due to the effect of VOC treatment (Figure 3). Additionally, evidence of the images is only colony growth changes of the fungal mycelium due to the effect of the volatile compounds. However, sporulation of the fungal growth was significantly decreased by the volatile organic compounds from all the yeast cultures, apart from *M.* aff. *pulcherrima* DN-HS isolate, as seen in Figure 3. Moreover, in the presence of *M*. *bullatus* DN-FY, *M*. aff. *pulcherrima* DN-MP and *M*. aff. *fructicola* 1-UDM, colonies of *A*. *flavus* were characterized by white mycelium. Additionally, compared with other yeast cultures, the most effective VOCs were produced by *M*. *bullatus* DN-FY, which mainly reduced sporulation. However, *M*. aff. *pulcherrima* DN-HS was not effective at inhibiting *A*. *flavus* production and showed similar morphological changes to the control plates.

### 2.4. Radial İnhibition of A. flavus

The yeasts that were most effective at inhibiting mycelial growth were *M.* aff. *pulcherrima* 32-AMM and 1-UDM. They were statistically different from other isolates and significantly reduced the mycelial growth of *A. flavus* (Table 4). The *M.* aff. *pulcherrima* DN-HS, *M.* aff. *pulcherrima* DN-UY and *M. pulcherrima* 26-BMD isolates promoted 100% inhibition after 3 days of incubation. All tested isolates significantly inhibited mycelial growth at three days of incubation compared with the other incubation periods (*p* < 0.05). For the eight isolates, fungal inhibition was observed within the range of 76.39–91.53% on day 7. Only *M.* aff. *pulcherrima* DN-UY mycelial growth inhibition rate was not significantly affected by the incubation period. Based on the findings, the yeast cultures were the same, but their antagonistic effects differed due to their isolation source; *M.* aff. *pulcherrima* 32-AMM was the most effective at inhibiting mycelial growth while *M.* aff. *pulcherrima* DN-MP was the least effective.

**Figure 4:**
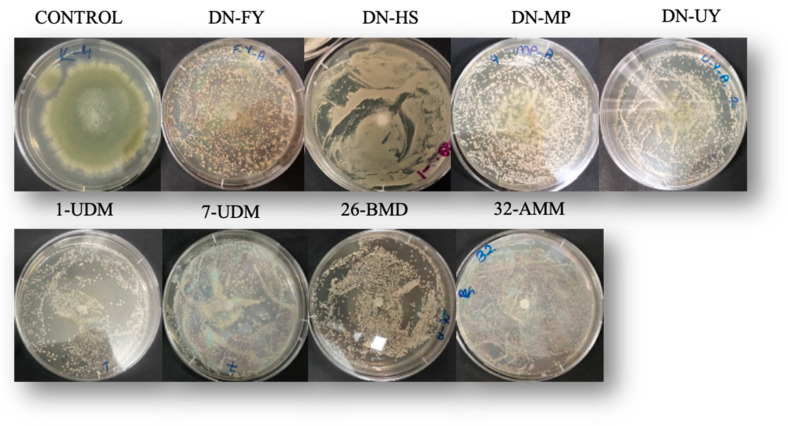
*A. flavus* spot inoculated with different yeasts on PDA at 25 °C for 7 days.

Apart from *M. bullatus* DN-FY, *M.* aff. *pulcherrima* DN-MP and *M. pulcherrima* 26-BMD, isolates did not exceed the spot inoculation area after 7 days of incubation (Figure 4).

### 2.5. Effect of Yeasts on In Vitro Aflatoxin B_1_ Production

Aflatoxin B_1_ production by *A. flavus* with the yeast interaction is shown in Table 5. *A. flavus* produced higher amounts of AFB_1_ without yeasts. *M. aff. pulcherrima* DN-HS was determined to be the most effective yeast for controlling AFB_1_ production (*p* < 0.05). When the yeast was in direct contact with fungal cultures, AFB_1_ production was nearly inhibited and VOCs of yeasts were ineffective at controlling AFB_1_ production. From this point of view, in vivo experiments on hazelnuts carried out using *M. aff. pulcherrima* DN-HS strain showed significant mycelial growth inhibition and the highest reduction in AFB_1_ production based on the in vitro assay, and was statistically significant compared with other yeast strains.

Based on the aflatoxin analysis results, only VOCs from *M. aff. fructicola* 1-UDM were effective at controlling AFB_1_ production by *A. flavus* (Table 6), while other isolates’ VOCs were either ineffective or promoted AFB_1_ production. VOCs from *M. aff. fructicola* 1-UDM reduced AFB_1_ production by 83%, 57% and 40% after 3, 5 and 7 days of incubation, respectively. The effects of VOCs on aflatoxin B_1_ were significantly decreased after three days of incubation. However, as can be seen from the results, there was no link between mycelium growth inhibition and AFB_1_ reduction. The average concentrations of AFB_1_ in the control plates (without exposure to VOCs) were 3040.89 ng/g, 4008.93 ng/g and 3265.02 ng/g after 3, 5 and 7 days of incubation, respectively. In control plates, aflatoxin B_1_ production was highest at 5 days of incubation at 25 °C. However, in aflatoxin B_1_ analysis, a higher standard deviation was observed from different replicates.

### 2.6. Biocontrol Activity of Antagonistic Yeasts on Hazelnuts

Based on the results of in vitro studies, *Metschnikowia* aff. *pulcherrima* DN-HS was selected to investigate the in vivo effect on *A. flavus* growth and its aflatoxin B_1_ production. The results of *Aspergillus flavus* counts and AFB_1_ levels on hazelnuts treated with *Metschnikowia* aff. *pulcherrima* DN-HS yeasts are shown in Table 7.

**Figure 5:**
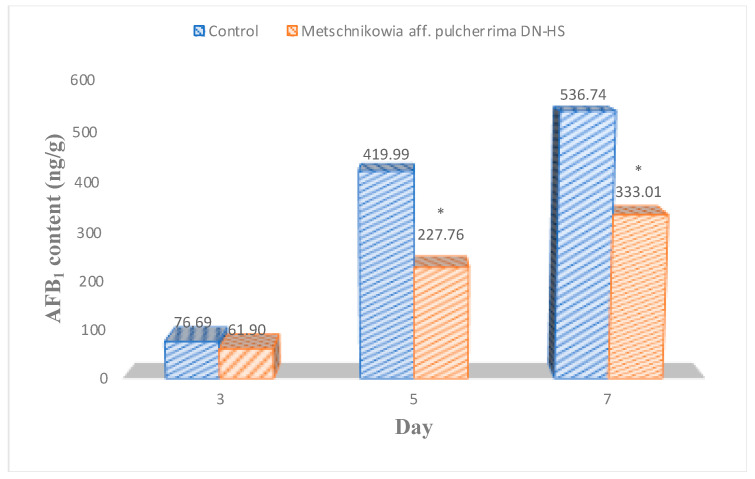
Hazelnut samples treated with *M.* aff. *pulcherrima* DN-HS; AFB1 content after different incubation periods. * Average values that are significantly different from the control based on the *t*-test (*p* < 0.05) are indicated by an asterisk in each column.

Compared with the control samples, antagonistic yeast treatments reduced *A. flavus* counts on hazelnuts by 46%, 51% and 35% after 3, 5 and 7 days of incubation, respectively. However, the effect of antagonistic yeast was highest on the 5th day of incubation. After 7 days of incubation, the samples treated with antagonists showed a slight difference in the fungal count compared with the control. The fungal counts were similar on the 5th and 7th days of incubation. A similar impact was shown in reducing AFB_1_ production. AFB_1_ content was significantly reduced by antagonistic yeasts at all incubation periods (Figure 5). Antagonistic yeast reduced AFB_1_ content on hazelnuts by 19, 46 and 38% at 3, 5 and 7 days. The antagonistic yeast was less effective at 7 days of incubation than at 5 days of incubation.

## 3. Discussion

In most cases, microbial antagonists are isolated from natural environments or the surfaces of living plant parts [16]. The study’s primary aim was to isolate and identify the yeast isolates from plant parts collected from Turkey to evaluate their antifungal and anti-aflatoxigenic activity through in vitro studies. Because of their biological and non-toxic properties, yeasts stand out among the microorganisms considered potential biological control agents [34]. Researchers have generally investigated the control of OTA production by microbial antagonists using in vitro and in vivo studies [21,23,46,47].

*Aspergillus flavus* causes grain degradation and yield loss. It is the main producer of aflatoxins with hepatotoxic, genotoxic, and teratogenic characteristics. High occurrence rates of aflatoxins were reported in rice, maize, nuts, wheat, cereals, dried fruits, and spices [48]. To our knowledge, this study is the first to report on yeasts of *Metschnikowia* sp., *Meyerozyma* sp. and *Moezymyces* (*Pseudozyma*) sp. with antagonistic activity against *A. flavus* and AFB_1_ production, although the biocontrol capabilities of bacterial species such as *Bacillus subtilis*, *Bacillus megaterium*, *Bacillus safensis*, and *Pseudomonas* spp. have been investigated under laboratory conditions [15,16,49]. However, these microorganisms were ineffective under field conditions [50]. Nowadays, to prevent aflatoxin production, non-aflatoxigenic *A. flavus* strains are used as antagonists [6]. Aflatoxin contamination was reduced by 70–90% in peanuts and cotton using non-toxigenic *Aspergillus* strains. However, researchers have also tried developing biocontrol agents for reducing aflatoxin contamination in the field [2].

A dual culture assay was initially used to screen yeast isolates’ antagonistic effect. The dual assay showed that all isolates of *M.* aff. *fructicola*, *M. pulcherrima*, *M. guilermondii* and *Moezymyces bullatus* have an antagonistic effect on the aflatoxin B_1_ producer *A. flavus*. These yeasts’ antagonistic effects against different fungal strains were documented by researchers [20,22,51,52,53]. Mycelial growth was restricted and yeast strains formed an inhibition zone in dual culture assays. In Israel, one strain of *M. fructicola* was registered to protect commercial postharvest fruits and vegetables [54]. It has been previously documented that the mechanism of action of *Metschnikowia* spp. is based on competition for nutrients such as iron, which is essential for fungal growth and depletion, and on the release of hydrolases [29]. Direct parasitism is an important component of the *Pseudozyma antarctica* biocontrol strategy [20]. Some *Pseudozyma* spp. exhibit antifungal activity due to the synthesis of ustilagic acid, a glycolipid active against various yeasts and yeast-like and filamentous fungi [55,56].

Numerous yeasts can produce volatile organic compounds (VOCs), and the volatiles have been linked to their antagonistic activity [23]. Several studies have been conducted on the production of VOCs by *M. pulcherrima* [14], *M. fructicola* [57] and *M. guilermondii* [58] against fungi. In this study, the highest antifungal activities of VOCs produced by *M. bullatus* DN-FY, *M.* aff. *pulcherrima* DN-MP and *M.* aff. *pulcherrima* 32-AMM were observed. On the other hand, *A. flavus* growth in the presence of some yeasts showed similarity with the absence of yeasts. Based on the literature review, there is no published information regarding aflatoxin B_1_ reduction by VOCs produced by yeasts. However, it has been widely reported that non-aflatoxigenic *Aspergillus* spp.’s VOCs are effective at reducing aflatoxin B_1_ production using the 2,3-dihydro-furan, trans-2- methyl-2-butenal, and 3-octanone [59]. Ethyl acetate [60] and ethyl alcohol [24] are the main VOCs that are naturally produced by *M. pullcherima*. The volatile antifungal metabolites produced by *M. guilermondii* include alcohols, aldehydes, hydrocarbons and terpenes [18]. Similar to our results, a study by Liu et al. [20] reported that *Metschnikowia citriensis* volatile organic compounds have more significant antagonistic activity against *P. Digitatum* and *Penicillium italicum*, with 3.17–11.36% of mycelium growth inhibition. Additionally, *Pseudozyma antarctica* VOCs showed a 5.38–7.47% reduction. Furthermore, in another study, *M. pulcherrima* VOCs reduced the mycelial growth of *A. carbonarius* by 6.5 ± 0.9% [60]. In a recent study, the same *M.* aff. *fructicola* 1-UDM isolate used in this study showed slightly higher mycelial growth inhibition against *P. digitatum* (28.44%) and *P. expansum* (19.88%) [41].

Similar to our findings, VOCs released by *Cyberlindnera jadinii*, *Candida friedrichii*, *Candida intermedia* and *Lachancea thermotolerans* prevent the sporulation of *A. carbonarius* [23]. Moreover, the noted inhibition of sporulation is consistent with the findings of Ul Hassan et al. [61] and Farbo et al. [23]. After a long incubation period, a reduction in mycelial growth inhibition was observed. This may be explained by a reduction in antifungal compounds. The reduction in volatile organic compounds emitted by *M. pulcherrima* was also reported after 5 days of incubation [62]. Additionally, Di Francesco et al. [26] stated that *Aeureobasidium pullulans* VOCs were emitted in the first 4 days of incubation and began to decrease after 4 days.

Studies on *Meyerozyma* sp. VOCs are limited and there are no studies on *Moezymyces* VOCs. In line with our findings, *M. guilliermondii* VOCs reduced mycelial growth of *P. expansum* by 13.67% and 18.9% at pH 4.5 [58]. Al-Maawali et al. [63] reported that VOCs produced by *M. guilermondii* reduced *Alternaria alternata* growth through production of tricosane and pentacosane.

The present study showed that *M.* aff. *fructicola* 1-UDM VOCs were the most effective yeasts for reducing mycelial growth and sporulation of *A. flavus*. AFB_1_ production by *A. flavus* MRC200744 was only reduced by VOCs of *M.* aff. *fructicola* 1-UDM. Similar to our results, despite the significant antagonistic effect on *A. flavus* growth, exposing the fungi to two *S. cerevisiae* strains did not show an effect on aflatoxin synthesis potential [61]. However, little information exists on *Moezmyces* (*Pseudozyma*) and *Meyerozyma* sp., and their mechanism of action should be investigated.

Different from the effect of VOCs, all studied isolates inhibited *A. flavus* growth and AFB_1_ production based on the spot inoculation method. *M.* aff. *fructicola* 1-UDM and *M.* aff. *pulcherrima* 32-AMM yeasts were the most effective at inhibiting mycelial growth. Similarly, Shude et al. [64] found that *Pseudozyma* sp. was effective at reducing *Fusarium gramineraum* mycelial growth and DON concentration. Oztekin and Karbancıoglu-Guler [60] documented that *M.* aff. *fructicola* 1-UDM and *M.* aff. *pulcherrima* 32-AMM had a strong antagonistic effect against *F. oxysporum*, *B. cinerea*, *P. digitatum*, *P. expansum* and *A. alternate*, similar to our findings.

Pawliowska et al. [65] stated that *Metschnikowia* sp. showed a strong antagonistic effect against *Alternaria*, *Botrytis*, *Fusarium* and *Rhizopus*. Reduction of some mycotoxins such as ochratoxin A [66] and patulin [67,68] by *Metschnikowia* sp. has been reported. Researchers have studied antagonistic yeasts in fruits such as apples [51], grapes [46] and strawberries [24] using in vivo studies. The effect of antagonistic microorganisms on aflatoxin production by *Bacillus* sp. in maize [15] and pistachio [69]; by aflasafe products in groundnuts and maize [3]; and by bacterial strains in corn [70] has been studied. Einloft et al. [15] reported that aflatoxin B_1_ production was reduced by 44.5–89.7% by different *Bacillus* sp. In another study, *B. amyloliquefaciens* and *B. subtilis* were found to be effective at reducing aflatoxin production on pistachio by *Aspergillus parasiticus* strain after 5 and 8 days of incubation. However, they reported that antagonists were ineffective at controlling aflatoxin after 12 days of incubation [69]. *Metschnikowia* aff. *pulcherrima* DN-HS strains grew faster than *Aspergillus flavus*. From this perspective, higher biocontrol efficiency was observed at 5 days, similar to the study by Siahmoshteh et al. [69] because yeast cultures reached a stationary phase while the fungus continued to proliferate. Additionally, different antagonistic bacteria inhibited *A. flavus* growth on pistachio by 45–70.5%. However, some of them were not effective at inhibiting the production of aflatoxin [70]. Our investigations showed that the direct reduction of *A. flavus* growth and its AFB_1_ production on hazelnuts using *Metschnikowia* sp. have not been previously reported.

## 4. Conclusions

Aflatoxin, which is produced predominantly by *Aspergillus flavus* and *Aspergillus parasiticus*, is one of the most important mycotoxins of concern in agriculture. Several food commodities have been infected at pre and post-harvest stages by this mycotoxin, including hazelnuts, pistachios, dried figs and maize. Generally, synthetic fungicides are used to control fungal growth and mycotoxin production. However, yeasts and bacteria have been extensively studied as biocontrol agents. For these reasons, in this study, antagonistic yeasts were isolated and shown to control *Aspergillus flavus* growth and aflatoxin B_1_ production. Furthermore, yeast cultures and their volatile organic compounds’ efficiency to control *Aspergillus flavus* growth and aflatoxin B_1_ production were investigated through in vitro studies. *A. flavus* growth and AFB_1_ production were significantly restricted by all tested yeasts that came in contact with the fungal strains. On the contrary, only *M.* aff. *fructicola* 1-UDM VOCs reduced AFB_1_ production. Based on the in vivo studies, *M.* aff. *pulcherrima* DN-HS was found to be effective at inhibiting *A. flavus* growth and AFB_1_ production on hazelnut samples. Future studies will address the increased in vitro efficacy of yeast against *A. flavus* growth and AFB_1_ production using preventative and curative treatments with practical spray applications. Furthermore, yeast strains might be combined with an agent to increase efficacy and the most crucial step, the hazelnut quality, might be investigated.

Our findings revealed that these species can also play an important role as biocontrol agents against *A. flavus* growth and AFB_1_ production on hazelnuts, and will be explored in further studies.

## 5. Materials and Methods

### 5.1. Pathogen

*A. flavus* MRC 200744 (AFB_1_ producer) was obtained from TUBITAK MAM Food Institue Culture Collection, Turkey, on slant agar media. Mold culture was grown on potato dextrose agar (PDA, Merck, Darmstad, Germany) at 25 °C; their stock solutions were prepared in 20% (*v*/*v*) glycerol and subsequently kept at −80 °C for antagonism tests.

### 5.2. Isolation of Yeast Cultures

The yeasts from grapes and blackberries were isolated and identified by Oztekin and Karbancıoğlu-Güler [42]. The yeasts were isolated from hawthorn, bean leaves, corn tassel and hoşkıran collected from Ordu/Turkey. The samples were kept in polyethylene bags and stored at −80 °C until yeast isolation. Approximately 5 g of the sample was incubated in an Erlenmeyer flask on a rotary shaker at 150 rpm in 50 mL of Tryptic Soy Broth (TSB) at 25 °C for 2–3 days. Following the incubation, decimal solutions were prepared with 0.85% saline solution, and 100 µL of the medium was then spread over malt extract agar (MEA) supplemented with 0.01% chloramphenicol. After incubation at 25 °C, single-cell colonies of the cultures were obtained from the MEA using the streak plate method, with 0.01% chloramphenicol, and incubated at 25 °C for 3 days. The colonies’ cell morphology was observed under a light microscope. Each yeast culture was kept at 4 °C in yeast extract peptone dextrose (YEPD) agar medium (10 g/L yeast extract, 20 g/L, peptone, 20 g/L glucose, 20 g/L agar) slant and 20% glycerol stock at −80 °C until further analysis.

### 5.3. Identification of Yeasts 

Yeasts were identified by sequencing the universal ITS1-5.8S-ITS2 (ITS AB28: (5′-ATA TGC TTA AGT TCA GCG GGT-3′) and ITS-TW81 (5′-GTT TCC GTA GGT GAA CCT GC-3′) primers. The primers were obtained from Sentiolab, Turkey. DNA extraction was performed using the Intron G-Spin DNA extraction kit (catalogue number 17045).

PCR analysis was conducted using 5 μL of Mg free Taq DNA polymerase buffer, 3 μL of MgCl_2_ (25 mM), 5 μL × 10 of deoxynucleotide triphosphates (2 mM each), 10 picomole/μL of each universal primer and 1.25 U Taq DNA polymerase (Thermo Scientific, EP0402, Vinius, Lithuania). PCR analysis was performed via the following steps: pre-denaturation for 5 min at 95 °C; 35 denaturation cycles carried out for 1 min at 95 °C; annealing was carried out for 1 min at a temperature suitable for each primer pair; extension was carried out for 1 min at 72 °C; and the final extension was carried out for 10 min at 72 °C. PCR products were separated by electrophoresis on agarose 3% (*w*/*v*) gels, photographed using a Vilber Lourmat Transilluminator, and purified using an Exo-SAP purification kit. DTCS cycle sequencing kit was used for DNA sequencing reactions and phylogenetic analysis. PCR products were analysed using ABI 3500 XL Genetic Analyzer (Applied Biosystems, Carlsbad, CA, USA). In order to differentiate fungal species, forward and reverse sequences obtained from Sanger sequencing were compared with NCBI Blast and aligned in BioEdit sequence alignment editor 7.2.5 software. Phylogenetic distances were computed using the Neighbour-joining method in MEGA-X version 10.2.2. The sequences obtained were compared to those included in the GenBank database using the Basic Local Alignment Search Tool (BLAST, http://www.ncbi.nlm.nih.gov (accessed on 11 September 2021)). The GenBank accession numbers of the sequences generated in this study are shown in Table 1.

### 5.4. In Vitro Assays

#### 5.4.1. Antagonistic Activity on Agar Plates

The mold strain *A. flavus* MRC 200744 was grown on PDA for 5–7 days at 25 °C. A pure suspension of *A. flavus* was then harvested in Ringer’s solution supplemented with 0.1% Tween 80 from PDA. The spore suspension was counted and adjusted to the desired concentration of 1 × 10^8^ spores/mL using a haemocytometer.

The yeasts were grown on YEPD agar (10 g/L yeast extract, 20 g/L, peptone, 20 g/L glucose, 20 g/L agar) slant and preserved at 4 °C until use. Yeast cultures were grown on YEPD broth (10 g/L yeast extract, 20 g/L, peptone, 20 g/L glucose) and incubated at 25 °C for 2 days before analysis. The interaction between yeast and *A. flavus* was evaluated using the dual culture method with minor modifications [21]. First, 100 µL of yeast suspension (1 × 10^7^ cells/mL) was streaked as a line 3 cm away from the PDA agar Petri plates edge (90 mm). After 48 h of incubation at 25 °C, 5 µL (10^5^ conidia/mL) of the spore suspension of *A. flavus* was spot inoculated 30 mm away from the yeast inoculation. Control Petri dishes were only inoculated with *A. flavus* spore suspension. The colony diameter on control plates reached 30 mm after 7 days of incubation at 25 °C.

#### 5.4.2. Effects of Volatile Organic Compounds (VOC)

A double Petri dish assay was used to investigate the efficacy of VOCs from each yeast against *A. flavus* as described by Ruiz Mayona et al. [24] and Parafati et al. [14], with slight modifications. Aliquots of 100 µL of yeast suspensions (1 × 10^7^ cell/mL) were spread on Petri plates containing YEPD agar and incubated for 48 h at 25 °C. Aliquots of 5 µL spore suspension (1 × 10^6^ spore/mL) were inoculated on the centre of the PDA Petri plates and dried at room temperature. Double dish assay was performed without inoculation of yeast for control plates. The lids of the petri dishes were then removed and the plates were covered and sealed with parafilm to avoid air leakage and incubated at 25 °C for 7 days. The diameter of the mycelia was measured daily. Reduction in *A. flavus* radial growth was calculated after 7 days. All analyses for each isolate were performed in quadruplicate and repeated three times.

#### 5.4.3. Radial Inhibition Assay of *A. flavus*

The antagonistic potential of yeast cultures against *A. flavus* was assessed using an agar plate inhibition assay as described by Medina-Córdova et al. [19]. A total of 100 µL of 1 × 10^7^ cells/mL suspension of yeast isolates was seeded on the PDA plates. After drying at room temperature, aliquots of 10 µL of the spore suspension (1 × 10^6^ cells/mL) were pipetted onto the centre of PDA plates and incubated for 7 days at 25 °C. The diameter of each colony was measured to calculate the radial inhibition (RI). The radial inhibition rate (RI) was calculated as follows:

RI (%) = [(C − T)/C] × 100 (1)

where C (control) was the average diameter of fungal colonies in the absence of yeast isolates and T (treatment) was the average diameter of fungal colonies in the presence of yeast. Each treatment had four replicates and was performed three times.

#### 5.4.4. Aflatoxin B_1_ Extraction from Medium

The effects of yeasts and their VOCs on aflatoxin B_1_ production were analyzed and plates were prepared as indicated in Section 2.5 and Section 2.6. In a preliminary experiment, a time course of mycelium growth and AFB_1_ production by *A. flavus* cultured on PDA at 25 °C was assessed by evaluating the radial growth and analyzing 3 plugs cut from duplicate colonies at 3, 5 and 7 days, and repeated twice. Aflatoxin B_1_ production by *A. flavus* in the presence of yeast was evaluated after 3, 5 and 7 days of incubation, as described by Bragulat et al. [71] and Farbo et al. [23]. Three agar plugs (6 mm in diameter) from the medium were removed with a sterile cork borer across the diameter of the agar plug in this approach. Samples were weighed and transferred to a 2.0 mL Eppendorf tube, 1.5 mL of methanol was added and the tubes were shaken slowly. After that, tubes were kept at room temperature for 1 h and centrifuged at 10,000 rpm for 5 min. The supernatants were transferred into a 0.22 µm centrifuge tube filter (Costar, Spin-X, Corning Incorporated, Corning, NY, USA) and again centrifuged at 10,000 rpm for 5 min. The supernatants were then transferred to a vial and preserved at 4 °C prior to analysis.

### 5.5. In Vivo Assays

#### 5.5.1. Sample Preparation

In vivo biocontrol ability of Metschnikowia aff. pulcherrima DN-HS, the most effective isolate for reducing aflatoxin B_1_ production in in vitro studies, was investigated on hazelnuts. Raw hazelnuts, supplied from Gürsoy A.Ş. (Ordu, Turkey), were vacuum-packed in non-permeable polyethylene bags and stored at −20 °C before analysis. First, hazelnut samples were rinsed with tap water. Then seeds were disinfected with sodium hypochloride for 2 min. Excess sodium hyphochloride was removed by rinsing with sterile distilled water and the seeds were finally dried under a laminar flow cabinet for several hours [19]. Sterilized hazelnut samples (50 g) were immersed for 30 min in yeast suspension (1 × 10^8^ cells/mL) under constant agitation. The control samples were immersed in the solution. Then hazelnut samples were dried. Finally, hazelnut samples were dipped into spore suspension 1 × 10^6^ spores/mL for 5 min and dried under a laminar flow cabinet. After that, the samples were incubated at 25 °C for 3, 5 and 7 days. All analyses were performed in triplicate.

#### 5.5.2. Effect of Antagonistic Yeast against *A. flavus* Growth on Hazelnuts

After each incubation period, 10 g of hazelnut samples were diluted and shaken in 90 mL of 0.1% Tween 80 at 200 rpm for 6 h [37] and then plated on dichloran rose bengal chloramphenicol agar (DRBC, Merck 100466, Darmstad, Germany). The plates were incubated at 25 °C for 5 days. After the incubation period, fungal counts were determined.

#### 5.5.3. Aflatoxin B_1_ Extraction from Hazelnuts

For aflatoxin analysis, 25 g of inoculated samples were homogenized with 125 mL of methanol: water (60:40, *v*/*v*) and shaken for 30 min. The extract was filtered, and 5 mL of samples were taken and mixed with 25 mL of phosphate-buffered saline (PBS) solution and cleaned through an immunoaffinity column AflaTest (VICAM, Water-town, MA, USA). The column was washed with 10 mL of water, and aflatoxins were then eluted with 1 mL of methanol and 1 mL of water. The samples were kept at −20 °C before the HPLC analysis. 

### 5.6. Aflatoxin B_1_ Detection and Quantification

AFB_1_ content in the medium and hazelnuts was determined using High Performance Liquid Chromatography (HPLC). Reversed-phase HPLC analysis was performed on a 10AVP HPLC system (Shimadzu, Milan, Italy) equipped with an RF-10AXL fluorescence detector set at wavelengths 360 nm for excitation and 420 nm for emission (Shimadzu, Milan, Italy) using post-column derivatization (KobraCell, 100 mA) (R-Biopharm Rhone Ltd., Glasgow, UK) with an analytical column (ACE, C18 250 × 4.6 mm, 5 mm, 100 A). For AFB_1_ quantification, the mobile phase consisted of water/acetonitrile/methanol (6/2/3, *v*/*v*/*v*), 120 mg/L of potassium bromide and 350 µL /L of 4 M nitric acid. The constant flow and column temperature were set to 1 mL/min and 25 °C, respectively. A volume of 100 µL was injected to be analyzed. The AFB_1_ retention time was around 13–14 min, the target peak of the samples. Aflatoxin content was calculated from the calibration curves obtained using aflatoxin standard solutions. For AFB_1_, LOD = 0.004 μg/mL, LOQ = 0.013 μg/mL and the mean recovery was 93%.

### 5.7. Statistical Analysis

All experiments were analyzed using SPSS 22.0 (SPSS Inc., Chicago, IL, USA), with the expression mean ± standard deviation. Different yeast isolates were compared using a one-way analysis of variance (ANOVA) test. Additionally, Tukey’s multiple range test was used to assess separations and differences were considered significant at *p* < 0.05. *t*-test was used to determine differences between treatments on hazelnut samples.

## Figures and Tables

**Table 1:** Molecular identification of yeast isolates by comparing the genomic sequences in the GenBank database using the BLAST algorithm. Yeasts marked with * were isolated and identified in a previous study by Oztekin and Karbancioglu-Güler [42].

Isolate ID.	Species or Taxonomic Group	Genbank Accession Number
1-UDM *	*Metschnikowia* aff. *fructicola*	KU862650.1
7-BYMD *	*Meyerozyma guilliermondii*	MT645353.1
26-BMD *	*Metschnikowia pulcherrima*	MT505669.1
32-AMM	*Metschnikowia* aff. *pulcherrima*	JX188181.1
DN-HS	*Metschnikowia* aff. *pulcherrima*	CP034458.1
DN-FY	*Moesziomyces bullatus*	KY424447.1
DN-MP	*Metschnikowia* aff. *pulcherrima*	CP034458.1
DN-UY	*Metschnikowia* aff. *pulcherrima*	CP034458.1

**Table 2:** Biocontrol activity of eight yeast isolates against *A. flavus*.

Isolate	Dual Culture (mm)			
	Day 3	Day 5	Day 7	Day 9
*Meyerozyma guilliermondii* 7-BYMD	18.33 ± 0.58 ^bC^	25.83 ± 1.47 ^bB^	29.00 ± 0.00 ^bA^	29.50 ± 0.86 ^bA^
*Moesziomyces bullatus* DN-FY	14.67 ± 0.58 ^cB^	27.00 ± 1.87 ^bA^	28.14 ± 2.19 ^bcA^	29.5 ± 1.64 ^bA^
*Metschnikowia* aff. *pulcherrima* DN-HS	15.50 ± 0.71 ^cC^	26.00 ± 1.26 ^bB^	26.25 ± 0.96 ^bcB^	28.57± 2.23 ^bA^
*Metschnikowia* aff. *pulcherrima* DN-MP	15.33 ± 0.58 ^cB^	25.57 ± 1.81 ^bA^	27.43 ± 3.15 ^bcA^	29.86 ± 2.19 ^bA^
*Metschnikowia* aff. *pulcherrima* DN-UY	14.33 ± 0.58 ^cC^	26.43 ± 1.51 ^bB^	28.86 ± 2.27 ^bBA^	29.00 ± 1.83 ^bA^
*Metschnikowia* aff. *pulcherrima* 32-AMM	18.00 ± 1.41 ^bC^	22.00 ± 1.41 ^cB^	25.25 ± 2.22 ^cAB^	27.33 ± 3.08 ^bA^
*Metschnikowia pulcherrima* 26-BMD	19.33 ± 1.15 ^bB^	24.86 ± 2.54 ^bcA^	29.25 ± 0.96 ^bcA^	27.8 ± 2.2 ^bA^
*Metschnikowia* aff. *fructicola* 1-UDM	20.00 ± 1.41 ^bB^	25.00 ± 3.35 ^bcA^	26.5 ± 2.06 ^bcA^	28.25 ± 3.73 ^bA^
Control	23.67 ± 1.15 ^aD^	34.70 ± 3.09 ^aC^	40.14 ± 2.48 ^aB^	46.20 ± 4.76 ^aA^

a, b, c: Different letters indicate statistically significant differences for each incubation time (*p* > 0.05). A, B, C, D: Different letters indicate statistically significant differences for each isolate (*p* > 0.05).

**Table 3:** The effect of volatile organic compounds produced by eight antagonistic yeasts against *A. flavus* after 3, 5 and 7 days of incubation.

Isolate	Mycelial Growth Inhibition (%)		
	Day 3	Day 5	Day 7
*Meyerozyma guilliermondii* 7-BYMD	14.57 ± 1.45 ^bcA^	5.80 ± 0.63 ^eB^	2.62 ± 1.22 ^bcC^
*Moesziomyces bullatus* DN-FY	21.88 ± 4.97 ^aA^	16.01 ± 2.47 ^abAB^	12.49 ± 2.10 ^aB^
*Metschnikowia* aff. *pulcherrima* DN-HS	15.22 ± 5.87 ^bcA^	8.47 ± 0.57 ^deA^	0.00 ± 0.00 ^cB^
*Metschnikowia* aff. *pulcherrima* DN-MP	20.25 ± 3.11 ^abA^	13.90 ± 3.04 ^bcB^	11.80 ± 6.48 ^aB^
*Metschnikowia* aff. *pulcherrima* DN-UY	18.58 ± 2.57 ^abA^	10.59 ± 1.75 ^cdB^	8.82 ± 2.86 ^aB^
*Metschnikowia* aff. *pulcherrima* 32-AMM	15.88 ± 2.70 ^bcA^	11.49 ± 1.85 ^cdB^	11.07 ± 3.39 ^aB^
*Metschnikowia pulcherrima* 26-BMD	13.40 ± 3.88 ^cA^	7.84 ± 1.96 ^deB^	0.00 ± 0.00 ^cC^
*Metschnikowia* aff. *fructicola* 1-UDM	19.04 ± 3.61 ^abA^	18.89 ± 2.47 ^aA^	6.69 ± 2.68 ^abB^

a, b, c, d, e: Different letters indicate statistically significant differences for each incubation time (*p* > 0.05). A, B, C: Different letters indicate statistically significant differences for each isolate (*p* > 0.05).

**Table 4:** In vitro mycelial growth inhibition of eight yeast isolates against *A. flavus*.

Isolate	Mycelial Growth Inhibition (%)		
	Day 3	Day 5	Day 7
*Meyerozyma guilliermondii* 7-BYMD	97.46 ± 4.76 ^aA^	88.96 ± 2.5 ^bB^	87.94 ± 2.27 ^abB^
*Moesziomyces bullatus* DN-FY	86.67 ± 3.57 ^dA^	79.17 ± 3.77 ^cB^	76.39 ± 3.60 ^cB^
*Metschnikowia* aff. *pulcherrima* DN-HS	100.0 ± 0.0 ^aA^	89.25 ± 2.59 ^bB^	85.78 ± 4.33 ^abB^
*Metschnikowia* aff. *pulcherrima* DN-MP	89.52 ± 2.43 ^cdA^	87.69 ± 2.78 ^bA^	77.51 ± 3.60 ^cB^
*Metschnikowia* aff. *pulcherrima* DN-UY	100.00 ± 0.00 ^aA^	87.69 ± 4.78 ^bB^	86.91 ± 4.00 ^abB^
*Metschnikowia* aff. *pulcherrima* 32-AMM	95.17 ± 5.39 ^abA^	90.36 ± 3.38 ^bA^	91.53 ± 2.90 ^aA^
*Metschnikowia pulcherrima* 26-BMD	100.00 ± 0.00 ^aA^	98.18 ± 4.45 ^aA^	80.98 ± 6.29 ^bcB^
*Metschnikowia* aff. *fructicola* 1-UDM	91.93 ± 2.05 ^bcA^	89.25 ± 3.67 ^bA^	88.85 ± 4.06 ^aA^

a, b, c, d: Different letters indicate statistically significant differences for each incubation time (*p* > 0.05). A, B: Different letters indicate statistically significant differences for each isolate (*p* > 0.05).

**Table 5:** Effects of yeasts on AFB_1_ production after 3, 5 and 7 days of incubation at 25 °C.

Isolate	AFB_1_ (ng/g)		
	Day 3	Day 5	Day 7
Control	2203.98 ± 57.17	2127.87 ± 158.43	1773.26 ± 11.40
*Meyerozyma guilliermondii* 7-BYMD	3.34 ± 6.68 ^ab^	1.96 ± 1.37 ^bc^	1.26 ± 0.00 ^b^
*Moesziomyces bullatus* DN-FY	3.81 ± 2.12 ^ab^	4.78 ± 7.24 ^b^	7.97 ± 7.4 ^ab^
*Metschnikowia* aff. *pulcherrima* DN-HS	ND ± 0.00 ^d^	12.9 ± 0.02 ^a^	10.95 ± 5.72 ^a^
*Metschnikowia* aff. *pulcherrima* DN-MP	1.92 ± 0.46 ^bc^	4.70 ± 1.72 ^b^	5.26 ± 4.53 ^ab^
*Metschnikowia* aff. *pulcherrima* DN-UY	3.12 ± 0.29 ^ab^	4.70 ± 3.14 ^b^	1.26 ± 0.04 ^b^
*Metschnikowia* aff. *pulcherrima* 32-AMM	3.18 ± 0.52 ^ab^	5.39 ± 1.78 ^b^	5.15 ± 1.91 ^ab^
*Metschnikowia pulcherrima* 26-BMD	0.98 ± 0.04 ^cd^	1.05 ± 0.73 ^c^	2.79 ± 0.15 ^ab^
*Metschnikowia* aff. *fructicola* 1-UDM	5.28 ± 0.26 ^a^	2.39 ± 1.58 ^bc^	3.61 ± 0.12 ^ab^

a, b, c, d: Different letters indicate statistically significant differences for each incubation time (*p* > 0.05).

**Table 6:** Effects of *M.* aff. *fructicola* 1-UDM VOC’s impact on AFB_1_ production and colony diameter of *A. flavus* after 3, 5 and 7 days of incubation at 25 °C.

Days	Colony Diameter (mm)		AFB_1_ (ng/g)	
	Control	1-UDM	Control	1-UDM
3	39.0 ± 3.22	30.8 ± 1.3	3040.89 ± 830.25 ^aA^	516.85 ± 0.00 ^bB^
5	55.5 ± 5.8	52.6 ± 5.7	4008.93 ± 1860 ^aA^	1708.633 ± 217.08 ^bA^
7	72.5 ± 2.9	68.7 ± 4.4	3265.02 ± 787 ^aA^	1954.88 ± 1594.27 ^bA^

a, b: Means of AFB_1_ content within each column followed by the same superscript letters are not significantly different (*p* > 0.05). A, B: Means of AFB_1_ content within each incubation time followed by the same superscript letters are not significantly different (*p* > 0.05).

**Table 7:** Effect of antagonistic yeast on *Aspergillus flavus* growth on hazelnuts after 3, 5 and 7 days of incubation.

	Fungal Counts (CFU/g)		
	Day 3	Day 5	Day 7
Control	4.63 ± 0.13	6.64 ± 0.01	6.72 ± 0.1
*Metschnikowia* aff. *pulcherrima* DN-HS	2.59 ± 0.10 *	5.79 ± 0.11 *	5.97 ± 0.39 *

* Average values significantly different from the control based on the *t*-test (*p* < 0.05) are indicated by an asterisk in each column.

## Data Availability

All the data generated for this study are included in the article.

## Supplementary Materials

The following supporting information can be downloaded at: https://www.mdpi.com/article/10.3390/toxins15060402/s1, Figure S1: In vitro test of antagonism of a yeast isolate against *A. flavus* using the dual culture technique on PDA plates after 3 (A), 5 (B) and 7 (C) days of incubation with the control and DN- HS yeast interaction.

## References

[1] Bhatnagar-Mathur P., Sunkara S., Bhatnagar-Panwar M., Waliyar F., Sharma K.K. (2015). Biotechnological Advances for Combating *Aspergillus flavus* and Aflatoxin Contamination in Crops. Plant Sci..

[2] Mwakinyali S.E., Ding X., Ming Z., Tong W., Zhang Q., Li P. (2019). Recent Development of Aflatoxin Contamination Biocontrol in Agricultural Products. Biol. Control.

[3] Agbetiameh D., Ortega-Beltran A., Awuah R.T., Atehnkeng J., Elzein A., Cotty P.J., Bandyopadhyay R. (2020). Field Efficacy of Two Atoxigenic Biocontrol Products for Mitigation of Aflatoxin Contamination in Maize and Groundnut in Ghana. Biol. Control.

[4] Accinelli C., Abbas H.K., Little N.S., Kotowicz J.K., Shier W.T. (2018). Biological Control of Aflatoxin Production in Corn Using Non-Aflatoxigenic *Aspergillus flavus* Administered as a Bioplastic-Based Seed Coating. Crop Prot..

[5] World Health Organization, International Agency for Research on Cancer (IARC) (1993). Some Naturally Occurring Substances: Food Items and Constituents, Heterocyclic Aromatic Amines and Mycotoxins. Monographs on the Evaluation of Carcinogenic Risks to Humans.

[6] Alaniz Zanon M.S., Clemente M.P., Chulze S.N. (2018). Characterization and Competitive Ability of Non-Aflatoxigenic *Aspergillus flavus* Isolated from the Maize Agro-Ecosystem in Argentina as Potential Aflatoxin Biocontrol Agents. Int. J. Food Microbiol..

[7] Kabak B. (2021). Aflatoxins in Foodstuffs: Occurrence and Risk Assessment in Turkey. J. Food Compos. Anal..

[8] European Food Safety Authority (2010). Management of Left-Censored Data in Dietary Exposure Assessment of Chemical Substances. EFSA J..

[9] Alasalvar C., Amaral J.S., Shahidi F. (2006). Functional Lipid Characteristics of Turkish Tombul Hazelnut (*Corylus avellana* L.). J. Agric. Food Chem..

[10] Salvatore M.M., Andolfi A., Nicoletti R. (2023). Mycotoxin Contamination in Hazelnut: Current Status, Analytical Strategies, and Future Prospects. Toxins.

[11] Kabak B. (2016). Aflatoxins in Hazelnuts and Dried Figs: Occurrence and Exposure Assessment. Food Chem..

[12] Nesci A., Passone M.A., Barra P., Girardi N., García D., Etcheverry M. (2016). Prevention of Aflatoxin Contamination in Stored Grains Using Chemical Strategies. Curr. Opin. Food Sci..

[13] Reddy K.R.N., Reddy C.S., Muralidharan K. (2009). Potential of Botanicals and Biocontrol Agents on Growth and Aflatoxin Production by *Aspergillus flavus* Infecting Rice Grains. Food Control.

[14] Parafati L., Vitale A., Restuccia C., Cirvilleri G. (2015). Biocontrol Ability and Action Mechanism of Food-Isolated Yeast Strains against Botrytis Cinerea Causing Post-Harvest Bunch Rot of Table Grape. Food Microbiol..

[15] Einloft T.C., Bolzan de Oliveira P., Radünz L.L., Dionello R.G. (2021). Biocontrol Capabilities of Three Bacillus Isolates towards Aflatoxin B1 Producer A. Flavus in vitro and on Maize Grains. Food Control.

[16] Kong Q., Shan S., Liu Q., Wang X., Yu F. (2010). Biocontrol of *Aspergillus flavus* on Peanut Kernels by Use of a Strain of Marine Bacillus Megaterium. Int. J. Food Microbiol..

[17] Accinelli C., Mencarelli M., Saccà M.L., Vicari A., Abbas H.K. (2012). Managing and Monitoring of *Aspergillus flavus* in Corn Using Bioplastic-Based Formulations. Crop Prot..

[18] Yan W., Gao H., Qian X., Jiang Y., Zhou J., Dong W., Xin F., Zhang W., Jiang M. (2021). Biotechnological Applications of the Non-Conventional Yeast Meyerozyma Guilliermondii. Biotechnol. Adv..

[19] Medina-Córdova N., López-Aguilar R., Ascencio F., Castellanos T., Campa-Córdova A.I., Angulo C. (2016). Biocontrol Activity of the Marine Yeast Debaryomyces Hansenii against Phytopathogenic Fungi and Its Ability to Inhibit Mycotoxins Production in Maize Grain (*Zea mays* L.). Biol. Control.

[20] Liu Y., Yao S., Deng L., Ming J., Zeng K. (2019). Different Mechanisms of Action of Isolated Epiphytic Yeasts against Penicillium Digitatum and Penicillium Italicum on Citrus Fruit. Postharvest Biol. Technol..

[21] Tryfinopoulou P., Chourdaki A., Nychas G.J.E., Panagou E.Z. (2020). Competitive Yeast Action against *Aspergillus carbonarius* Growth and Ochratoxin A Production. Int. J. Food Microbiol..

[22] Pantelides I.S., Christou O., Tsolakidou M.D., Tsaltas D., Ioannou N. (2015). Isolation, Identification and in vitro Screening of Grapevine Yeasts for the Control of Black Aspergilli on Grapes. Biol. Control.

[23] Farbo M.G., Urgeghe P.P., Fiori S., Marcello A., Oggiano S., Balmas V., Hassan Z.U., Jaoua S., Migheli Q. (2018). Effect of Yeast Volatile Organic Compounds on Ochratoxin A-Producing *Aspergillus carbonarius* and *A. Ochraceus*. Int. J. Food Microbiol..

[24] Ruiz-Moyano S., Hernández A., Galvan A.I., Córdoba M.G., Casquete R., Serradilla M.J., Martín A. (2020). Selection and Application of Antifungal VOCs-Producing Yeasts as Biocontrol Agents of Grey Mould in Fruits. Food Microbiol..

[25] Huang Y., Sun C., Guan X., Lian S., Li B., Wang C. (2021). Biocontrol Efficiency of Meyerozyma Guilliermondii Y-1 against Apple Postharvest Decay Caused by Botryosphaeria Dothidea and the Possible Mechanisms of Action. Int. J. Food Microbiol..

[26] Di Francesco A., Di Foggia M., Baraldi E. (2020). Aureobasidium Pullulans Volatile Organic Compounds as Alternative Postharvest Method to Control Brown Rot of Stone Fruits. Food Microbiol..

[27] Lino de Souza M., Silva Ribeiro L., Gabriela da Cruz Pedrozo Miguel M., Roberto Batista L., Freitas Schwan R., Henrique Medeiros F., Ferreira Silva C. (2021). Yeasts Prevent Ochratoxin A Contamination in Coffee by Displacing *Aspergillus carbonarius*. Biol. Control.

[28] Ponsone M.L., Nally M.C., Chiotta M.L., Combina M., Köhl J., Chulze S.N. (2016). Evaluation of the Effectiveness of Potential Biocontrol Yeasts against Black Sur Rot and Ochratoxin A Occurring under Greenhouse and Field Grape Production Conditions. Biol. Control.

[29] Saravanakumar D., Ciavorella A., Spadaro D., Garibaldi A., Gullino M.L. (2008). Metschnikowia Pulcherrima Strain MACH1 Outcompetes Botrytis Cinerea, Alternaria Alternata and Penicillium Expansum in Apples through Iron Depletion. Postharvest Biol. Technol..

[30] Zhimo V.Y., Kumar A., Biasi A., Salim S., Feygenberg O., Toamy M.A., Abdelfattaah A., Medina S., Freilich S., Wisniewski M. (2021). Compositional Shifts in the Strawberry Fruit Microbiome in Response to Near-Harvest Application of Metschnikowia Fructicola, a Yeast Biocontrol Agent. Postharvest Biol. Technol..

[31] Lahlali R., Hamadi Y., Drider R., Misson C., El Guilli M., Jijakli M.H. (2014). Control of Citrus Blue Mold by the Antagonist Yeast Pichia Guilliermondii Z1: Compatibility with Commercial Fruit Waxes and Putative Mechanisms of Action. Food Control.

[32] Yang Q., Ma J., Solairaj D., Fu Y., Zhang H. (2022). Efficacy of Meyerozyma Guilliermondii in Controlling Patulin Production by Penicillium Expansum in Shuijing Pears. Biol. Control.

[33] Wang Z., Li J., Liu J., Tian X., Zhang D., Wang Q. (2021). Management of Blue Mold (Penicillium Italicum) on Mandarin Fruit with a Combination of the Yeast, Meyerozyma Guilliermondii and an Alginate Oligosaccharide. Biol. Control.

[34] Öztekin S., Karbancioglu-Guler F. (2023). Biological Control of Green Mould on Mandarin Fruit through the Combined Use of Antagonistic Yeasts. Biol. Control.

[35] Millan A.F., Fernandez-irigoyen J., Larraya L., Farran I., Veramendi J. (2023). Metschnikowia Pulcherrima as an Efficient Biocontrol Agent of Botrytis Cinerea Infection in Apples: Unraveling Protection Mechanisms through Yeast Proteomics. Biol. Control.

[36] Cheng L., Zhou L., Li D., Gao Z., Teng J., Nie X., Guo F., Wang C., Wang X., Li S. (2023). Combining the Biocontrol Agent Meyerozyma Guilliermondii with UV-C Treatment to Manage Postharvest Gray Mold on Kiwifruit. Biol. Control.

[37] Parafati L., Restuccia C., Cirvilleri G. (2022). Efficacy and Mechanism of Action of Food Isolated Yeasts in the Control of *Aspergillus flavus* Growth on Pistachio Nuts. Food Microbiol..

[38] Sen Y., Onal-Ulusoy B., Mutlu M. (2019). *Aspergillus* Decontamination in Hazelnuts: Evaluation of Atmospheric and Low-Pressure Plasma Technology. Innov. Food Sci. Emerg. Technol..

[39] Dasan B.G., Boyaci I.H., Mutlu M. (2017). Nonthermal Plasma Treatment of *Aspergillus* spp. Spores on Hazelnuts in an Atmospheric Pressure Fluidized Bed Plasma System: Impact of Process Parameters and Surveillance of the Residual Viability of Spores. J. Food Eng..

[40] Sen Y., Onal-Ulusoy B., Mutlu M. (2019). Detoxification of Hazelnuts by Different Cold Plasmas and Gamma Irradiation Treatments. Innov. Food Sci. Emerg. Technol..

[41] Mošovská S., Medvecká V., Gregová M., Tomeková J., Valík Ľ., Mikulajová A., Zahoranová A. (2019). Plasma Inactivation of *Aspergillus flavus* on Hazelnut Surface in a Diffuse Barrier Discharge Using Different Working Gases. Food Control.

[42] Oztekin S., Karbancioglu-Guler F. (2021). Bioprospection of *Metschnikowia* sp. Isolates as Biocontrol Agents against Postharvest Fungal Decays on Lemons with Their Potential Modes of Action. Postharvest Biol. Technol..

[43] Saitou N., Nei M. (1987). The Neighbor-Joining Method: A New Method for Reconstructing Phylogenetic Trees. Mol. Biol. Evol..

[44] Tamura K., Nei M., Kumar S. (2004). Prospects for Inferring Very Large Phylogenies by Using the Neighbor-Joining Method. Proc. Natl. Acad. Sci. USA.

[45] Kumar S., Stecher G., Li M., Knyaz C., Tamura K. (2018). MEGA X: Molecular Evolutionary Genetics Analysis across Computing Platforms. Mol. Biol. Evol..

[46] Zhu C., Shi J., Jiang C., Liu Y. (2015). Inhibition of the Growth and Ochratoxin A Production by *Aspergillus carbonarius* and *Aspergillus ochraceus* in vitro and in vivo through Antagonistic Yeasts. Food Control.

[47] Fiori S., Urgeghe P.P., Hammami W., Razzu S., Jaoua S., Migheli Q. (2014). Biocontrol Activity of Four Non- and Low-Fermenting Yeast Strains against *Aspergillus carbonarius* and Their Ability to Remove Ochratoxin A from Grape Juice. Int. J. Food Microbiol..

[48] Rushing B.R., Selim M.I. (2019). Aflatoxin B1: A Review on Metabolism, Toxicity, Occurrence in Food, Occupational Exposure, and Detoxification Methods. Food Chem. Toxicol..

[49] Palumbo J.D., Baker J.L., Mahoney N.E. (2006). Isolation of Bacterial Antagonists of *Aspergillus flavus* from Almonds. Microb. Ecol..

[50] Dorner J.W. (2004). Biological Control of Aflatoxin Contamination of Crops. J. Toxicol. Toxin Rev..

[51] Spadaro D., Lorè A., Garibaldi A., Gullino M.L. (2013). A New Strain of Metschnikowia Fructicola for Postharvest Control of Penicillium Expansum and Patulin Accumulation on Four Cultivars of Apple. Postharvest Biol. Technol..

[52] Rodriguez Assaf L.A., Pedrozo L.P., Nally M.C., Pesce V.M., Toro M.E., Castellanos de Figueroa L.I., Vazquez F. (2020). Use of Yeasts from Different Environments for the Control of Penicillium Expansum on Table Grapes at Storage Temperature. Int. J. Food Microbiol..

[53] Fernandez-San Millan A., Larraya L., Farran I., Ancin M., Veramendi J. (2021). Successful Biocontrol of Major Postharvest and Soil-Borne Plant Pathogenic Fungi by Antagonistic Yeasts. Biol. Control.

[54] Banani H., Spadaro D., Zhang D., Matic S., Garibaldi A., Gullino M.L. (2015). Postharvest Application of a Novel Chitinase Cloned from Metschnikowia Fructicola and Overexpressed in Pichia Pastoris to Control Brown Rot of Peaches. Int. J. Food Microbiol..

[55] Kulakovskaya T.V., Shashkov A.S., Kulakovskaya E.V., Golubev W.I. (2005). Ustilagic Acid Secretion by Pseudozyma Fusiformata Strains. FEMS Yeast Res..

[56] Golubev W.I., Kulakovskaya T.V., Shashkov A.S., Kulakovskaya E.V., Golubev N.V. (2008). Antifungal Cellobiose Lipid Secreted by the Epiphytic Yeast Pseudozyma Graminicola. Microbiology.

[57] Cordero-Bueso G., Mangieri N., Maghradze D., Foschino R., Valdetara F., Cantoral J.M., Vigentini I. (2017). Wild Grape-Associated Yeasts as Promising Biocontrol Agents against Vitis Vinifera Fungal Pathogens. Front. Microbiol..

[58] Agirman B., Erten H. (2020). Biocontrol Ability and Action Mechanisms of Aureobasidium Pullulans GE17 and Meyerozyma Guilliermondii KL3 against Penicillium Digitatum DSM2750 and Penicillium Expansum DSM62841 Causing Postharvest Diseases. Yeast.

[59] Moore G.G., Lebar M.D., Carter-Wientjes C.H., Gilbert M.K. (2021). The Potential Role of Fungal Volatile Organic Compounds in *Aspergillus flavus* Biocontrol Efficacy. Biol. Control.

[60] Oro L., Feliziani E., Ciani M., Romanazzi G., Comitini F. (2018). Volatile Organic Compounds from Wickerhamomyces Anomalus, Metschnikowia Pulcherrima and Saccharomyces Cerevisiae Inhibit Growth of Decay Causing Fungi and Control Postharvest Diseases of Strawberries. Int. J. Food Microbiol..

[61] Ul Hassan Z., Al Thani R., Atia F.A., Alsafran M., Migheli Q., Jaoua S. (2021). Application of Yeasts and Yeast Derivatives for the Biological Control of Toxigenic Fungi and Their Toxic Metabolites. Environ. Technol. Innov..

[62] Contarino R., Brighina S., Fallico B., Cirvilleri G., Parafati L., Restuccia C. (2019). Volatile Organic Compounds (VOCs) Produced by Biocontrol Yeasts. J. Food Microbiol..

[63] Al-Maawali S.S., Al-Sadi A.M., Ali Khalifa Alsheriqi S., Nasser Al-Sabahi J., Velazhahan R. (2021). The Potential of Antagonistic Yeasts and Bacteria from Tomato Phyllosphere and Fructoplane in the Control of Alternaria Fruit Rot of Tomato. All Life.

[64] Shude S.P.N., Mbili N.C., Yobo K.S. (2021). Epiphytic Yeasts as Potential Antagonists against Fusarium Head Blight of Wheat (*Triticum aestivum* L.) Caused by Fusarium Graminearum Sensu Strict. J. Saudi Soc. Agric. Sci..

[65] Pawlikowska E., James S.A., Breierova E., Antolak H., Kregiel D. (2019). Biocontrol Capability of Local *Metschnikowia* sp. Isolates. Antonie Van Leeuwenhoek.

[66] Patharajan S., Reddy K.R.N., Karthikeyan V., Spadaro D., Lore A., Gullino M.L., Garibaldi A. (2011). Potential of Yeast Antagonists on Invitro Biodegradation of Ochratoxin A. Food Control.

[67] Spadaro D., Droby S. (2016). Development of Biocontrol Products for Postharvest Diseases of Fruit: The Importance of Elucidating the Mechanisms of Action of Yeast Antagonists. Trends Food Sci. Technol..

[68] Settier-Ramírez L., López-Carballo G., Hernández-Muñoz P., Fontana A., Strub C., Schorr-Galindo S. (2021). New Isolated *Metschnikowia pulcherrima* Strains from Apples for Postharvest Biocontrol of *Penicillium expansum* and Patulin Accumulation. Toxins.

[69] Siahmoshteh F., Siciliano I., Banani H., Hamidi-Esfahani Z., Razzaghi-Abyaneh M., Gullino M.L., Spadaro D. (2017). Efficacy of Bacillus Subtilis and Bacillus Amyloliquefaciens in the Control of Aspergillus Parasiticus Growth and Aflatoxins Production on Pistachio. Int. J. Food Microbiol..

[70] Zhang J., Wang Y., Du Z., Lin D., Huo L., Qin L., Wang W., Qiang L., Yao Y., An Y. (2021). Screening, Identification and Antagonistic Effect of Antagonistic Bacteria JTFM1001 against Aflatoxin Contamination in Corn. Oil Crop Sci..

[71] Bragulat M.R., Abarca M.L., Cabañes F.J. (2001). An Easy Screening Method for Fungi Producing Ochratoxin A in Pure Culture. Int. J. Food Microbiol..

