# Supplementary material for: Biocontrol Potential of Antagonistic Yeasts on In Vitro and In Vivo Aspergillus Growth and Its AFB1 Production
Source: Toxins (Basel). 2023 Jun 19;15(6):402. doi: 10.3390/toxins15060402 (PMC10304067; doi:10.3390/toxins15060402)
Supplement: Supplementary file 1 [file toxins-15-00402-s001.zip › toxins-2427712-supplementary.pdf]

Supplementary Materials: Biocontrol Potential of Antagonistic Yeasts on In Vitro and In Vivo *Aspergillus* Growth and Its AFB_1 Production

Dilara Nur Dikmetas, Hayrettin Özer and Funda Karbancıoglu-Guler

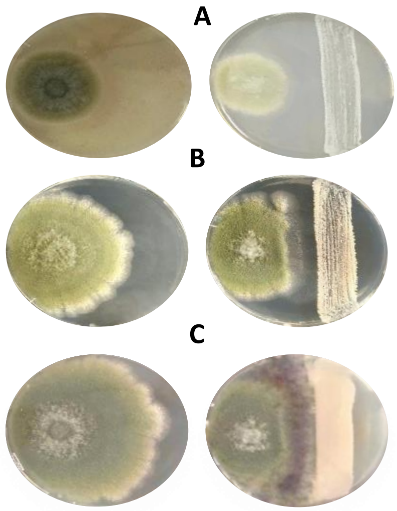

Figure S1. *In vitro* test of antagonism of a yeast isolate against *A. flavus* using the dual culture technique on PDA plates after 3 (**A**), 5 (**B**) and 7 (**C**) days of incubation with the control and DN-HS yeast interaction.
